# The m^6^A reader YTHDF3 promotes TNBC progression by regulating CENPI stabilization

**DOI:** 10.3389/fonc.2025.1546723

**Published:** 2025-05-08

**Authors:** Yulu Zhang, Shunji Chen, Qiaosheng Wu

**Affiliations:** ^1^ Department of Breast Surgery, Third Hospital of Nanchang, Nanchang, Jiangxi, China; ^2^ Department of Orthopedics, Third Hospital of Nanchang, Nanchang, Jiangxi, China

**Keywords:** TNBC, YTHDF3, CENPI, m^6^A modification, prognostic factors

## Abstract

**Background:**

RNA *N* ^6^-methyladenosine (m^6^A) readers mediate cancer progression. However, the role of eiptranscriptomic modifications such as m^6^A in the regulation of TNBC progression is unclear.

**Methods:**

High-throughput library screening identifies the key m^6^A regulator YTHDF3 in TNBC. Cell and animal experiments were used to identify that YTHDF3 promoted TNBC tumorigenesis to enhance Centromere protein I (CENPI) translation via m^6^A modification.

**Results:**

We showed that the *N* ^6^-methyladenosine (m^6^A) reader YTHDF3 was an independent risk factor in TNBC and was associated with poor prognosis of patients. Notedly, overexpression YTHDF3 promoted TNBC tumorigenesis in an m^6^A modification, while TNBC knockdown markedly inhibited proliferation and migratory ability of tumor cells *in vitro* and in vivo. Mechanistically, Mechanistically, YTHDF3 interacted with Centromere protein I (CENPI) mRNAs to prolong stability of m^6^A-modified RNA.

**Conclusion:**

Our findings indicated that m^6^A reader YTHDF3 contributed to tumorigenesis and poor prognosis, providing a potential prognostic biomarker and therapeutic target for TNBC.

## Introduction

Triple-negative breast cancer (TNBC) is defined as the absence of estrogen receptor (ER), progesterone receptor (PR), and human epidermal growth factor receptor 2 (HER2) (1, 2). Compared to other subtypes, TNBC represents the most challenging subtype of breast cancer owing to its highly invasive nature, distant metastasis and recurrence, and lack of targeted therapy options (3). Therefore, it is imperative to further illustrate the molecular pathogenesis of TNBC to develop novel therapeutic strategies.

RNA *N*(6)-methyladenosine (m^6^A) modification is one of the most pervasive and abundant RNA modifications and plays a critical role in regulating mRNA stability, splicing, transport, localization, and translation. m^6^A modifications are dynamic and reversible posttranscriptional RNA modifications that are mediated by m^6^A regulators, such as methyltransferases (“writers”: METTL3, METTL14, and WTAP), demethylases (“erasers”: ALKBH5 and FTO), and m^6^A-binding proteins (“readers”: YTHDs and IGF2BPs) (4, 5). Recent studies have shown that the dysregulation of RNA m^6^A methyltransferases/demethylases plays a crucial role in cancer initiation, progression, and metastasis by modulating the oncogenic mRNA stability (6, 7). To date, the role of various m^6^A reader proteins in the progression of TNBC remains largely unexplored. Therefore, it is necessary to elucidate the biological importance of m^6^A regulators in promoting tumors.

Centromere protein I (CENPI) is an important member of the centromeric protein family, which is crucial to chromosome alignment and segregation (8). Genetic mutations in CENPI can lead to centromere instability, as evidence-based studies show dynamic centromere break/deletion/isochromosome/translocation formation (9). CENPI mutations can be detected in some human cancers, such as adrenocortical carcinoma, lung adenocarcinoma (8), breast cancer (10), and colorectal cancer (CRC) (11). These findings have suggested that CENPI acts through distinct mechanisms in different tumor types.

Here, we demonstrated that a critical m^6^A reader, YTH domain-containing family protein 3 (YTHDF3), directly recognized m^6^A‐modified mRNAs of the CENPI gene and stabilized CENPI mRNAs to contribute to tumorigenesis in TNBC. In summary, our results revealed that m^6^A modulates cancer cell plasticity and provides potential therapeutic targets for TNBC.

## Materials and methods

### Data collection

mRNA-seq datasets and clinical information on TNBC were obtained from The Cancer Genome Atlas (TCGA) database (https://cancergenome.nih.gov/).

### Survival analysis and Cox regression analysis

The difference in overall survival (OS) between the two groups was evaluated by the Kaplan–Meier (K-M) survival analysis using the log-rank test. Cox regression analysis and hazard ratio (HR) were performed using the R package “survival”.

### DEG analysis

The “limma” package of the R software was used to identify the differentially expressed genes (DEGs) between high-YTHDF3 and low-YTHDF3 expression groups. The top 1,500 upregulated genes were screened.

### Construction of a protein–protein interaction network

The mRNAs were included in a protein–protein interaction (PPI) network using the STRING database (https://string-db.org/) with a confidence score of >0.8. Cytoscape (version 3.8.1) was used to visualize the PPI network (12).

### Gene set variation analysis

Gene set variation analysis (GSVA) was used to investigate the variation in biological processes between different cuproptosis regulation patterns with the R package “GSVA”.

### Cell lines and cell culture

Human TNBC cell lines (MDA-MB-231 and SUM159PT) were purchased from the Chinese Academy of Sciences Cell Bank. These cell lines were grown on dishes in DMEM/F12 (Gibco, Grand Island, NY, USA) supplemented with 10% Gibco fetal bovine serum (FBS). All cells were seeded into culture dishes and incubated at 37°C in a 5% CO_2_ humidified incubator.

### Cell proliferation assay

Cell Counting Kit-8 (CCK-8) assay was used to assess tumor cell viability following the manufacturer’s instructions. TNBC cells (2 × 10^3^ cells, 100 μL per well) were seeded into 96-well plates, and CCK-8 reagent (Bio-Techne, Hong Kong, China; Cat# 7368/5ML) was added at 24, 48, 72, and 96 h. The absorbance at 450 nm was measured using a microplate reader. For the colony formation assay, cells were seeded in 6-well plates at a density of 1 × 10^3^ cells per well and cultured for 7–10 days. The colonies were then stained with 0.2% crystal violet and documented.

### Transwell migratory experiment

To evaluate cell migratory ability, MDA-MB-231 and SUM159PT cells (3 × 10^4^) were seeded into the upper chamber and incubated overnight. Subsequently, 500 μL complete medium supplemented with 10% FBS was added into the lower chamber of the Transwell insert to promote cell migration. After 24 h, cells migrating through the membrane of Transwell inserts were stained with crystal violet and photographed by microscopy.

### Wound healing assay

Cells were seeded in a 6-well plate and incubated overnight. The next day, the inserts were removed, and serum-free medium was added. After 36 h, ImageJ was used to calculate the migratory rate.

### Western blotting

Cells and tissue were lysed using Radio Immuno Precipitation Assay (RIPA) lysis (Thermo Fisher Scientific, Waltham, MA, USA; Cat# 89900) buffer supplemented with protease and phosphatase inhibitors (Beyotime, Shanghai, China). Protein concentrations were determined using the BCA kit (Thermo Fisher Scientific; Cat# 23225). Lysates were then separated by Sodium Dodecyl Sulfate Polyacrylamide Gel Electrophoresis (SDS–PAGE) gels and transferred onto polyvinylidene difluoride (PVDF) membranes. Membranes were incubated overnight at 4°C with specific primary antibodies. After washing, membranes were incubated with secondary antibodies at room temperature for 2 h.

### Chemical reagents, antibodies, and transfection

HUR (RiboBio, Guangzhou, China), actinomycin D (APExBio, Houston, TX, USA), anti‐YTHDF3 (Proteintech, Wuhan, China; Cat# 25537-1-AP), anti‐CENPI (Abcam, Cambridge, UK; Cat# ab168778), anti‐GAPDH (Proteintech; Cat# 60004-1-Ig), and m^6^A (Proteintech; Cat# 68055-1-Ig) were used.

YTHDF3 overexpression plasmids and short hairpin RNA (shRNA) were purchased from GeneChem (Shanghai, China). The specific human shRNA sequences were shYTHDF3#1, GGACGTGTGTTTATAATTA; shYTHDF3#2, GACTAGCATTGCAACCAAT. All transfections were performed according to the manufacturers’ instructions.

### mRNA stability assay

To explore the stability of CENPI mRNA and protein under the downregulation or upregulation of YTHDF3, cells were treated with 10 µm actinomycin D for 0, 3, and 6 h prior to RNA extraction. The procedures of total RNA isolation and RT–qPCR were performed as described above. The transcript level of CENPI mRNA was estimated as the half-life of the mRNA and normalized to GAPDH as the standard.

### Quantitative RT–PCR

Total RNA was extracted from cells using TRIzol reagent (Invitrogen, Carlsbad, CA, USA; Cat# 15596026CN) according to the manufacturer’s instructions. For reverse transcription, 1 μg of RNA was applied (Cat# R323-01; Vazyme, Nanjing, China). Real-time PCR analysis was performed using SYBR Premix Ex TaqTM (Tli RNaseH Plus) (TaKaRa, Dalian, China). The amplification primers were as follows: (YTHDF1 forward: ACCTGTCCAGCTATTACCCG; YTHDF1 reverse: TGGTGAGGTATGGAATCGGAG) (YTHDF2 forward: CCTTAGGTGGAGCCATGATTG; YTHDF2 reverse: TCTGTGCTACCCAACTTCAGT) (YTHDF3 forward: TCAGAGTAACAGCTATCCACCA; YTHDF3 reverse: GGTTGTCAGATATGGCATAGGCT).

### RNA immunoprecipitation assay

The Geneseed RIP Kit (MBL International, Schaumburg, IL, USA; Cat# RN1001) was performed for the RNA immunoprecipitation (RIP) assay according to the instructions. Briefly, magnetic beads were mixed with anti-m^6^A/YTHDF3/IgG antibodies, and then cell lysates were added. Next, the bound complexes were thoroughly washed, eluted, purified, and analyzed by RT–qPCR. Enrichment of precipitated RNAs was normalized relative to input controls.

### Tumor xenograft model

Animal experiments are approved by the ethics committee and carried out in strict accordance with the requirements. Male BALB/c nude mice (4–6 weeks, 18–22 g) purchased from the Animal Center of Biotechnology Co., Ltd (Beijing, China) were maintained under specific pathogen-free conditions. Treated SUM159PT cell suspensions (1 × 10^6^ cells) were mixed 1:1 and injected subcutaneously into the right axillae of nude mice. The quantification of immunohistochemical staining was performed using the Image-Pro Plus 6.0 software. Tumor volume was calculated as follows: (longest diameter) × (shortest diameter)2 × (π/6). All animal experimental procedures used in this study were approved by the Animal Ethics Committee of the Third Hospital of Nanchang. All methods were performed in accordance with the Animal Research: Reporting of In Vivo Experiments (ARRIVE) guidelines and related guidelines and regulations.

### Statistical analysis

All data were presented as means and standard deviations (SDs). Differences between the two independent groups were determined using a two-tailed Student’s t-test. A one-way ANOVA test was performed for multiple comparisons. Statistical analysis was performed using the GraphPad Prism 9 software. A p-value less than 0.05 was considered statistically significant.

## Results

### YTHDF3 was identified as a core m^6^A regulator in TNBC

**Figure 1:**
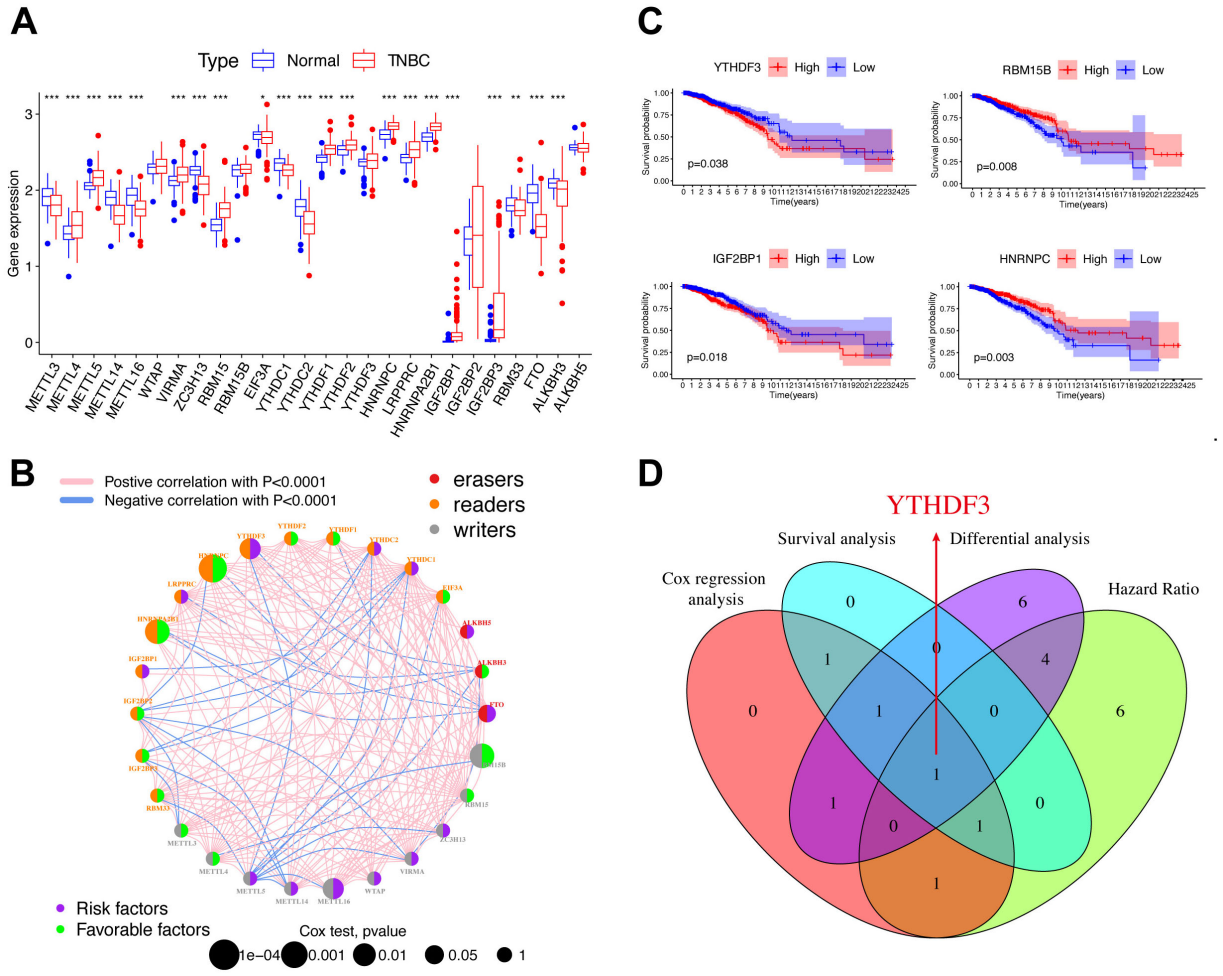
YTHDF3 was identified as a core m^6^A regulator in TNBC. **(A)** Differential expression of m^6^A regulators between normal and TNBC tissues in TCGA cohort (normal, blue; TNBC, red). Significant results are indicated as ***p < 0.001, **p < 0.01, and *p < 0.05. **(B)** The interaction of expression on m^6^A regulators in TNBC. Different biological functions of m^6^A regulators were depicted by circles in different colors. The circle size represented the effect of each regulator on the prognosis by p-value. The lines linking regulators represent their interactions, pink represents positive correlation, and blue represents negative correlation. Green dots in the circle show favorable factors of prognosis. Purple dots in the circle show risk factors of prognosis. **(C)** Kaplan–Meier survival analysis showed that indicated genes (YTHDF3, IGF2BP1, RBM15B, and HNRNPC) exhibited prognosis in TNBC patients based on TCGA data. **(D)** The Venn plot shows that YTHDF3 was identified based on the intersection analysis. Cox, p < 0.05; HR > 1; differential analysis between normal and TNBC in TCGA database; the cutoff criteria were set aslog2 fold change (FC)|> 0 and p < 0.05. TNBC, triple-negative breast cancer; TCGA, The Cancer Genome Atlas; HR, hazard ratio.

m^6^A, as an epigenetic modification, is dynamic and reversible, established mainly by three enzymes (**Figure 1A** and **Supplementary Table S1**), namely, m^6^A methyltransferases (also called writers: METTL3, METTL14, etc.), m^6^A demethylases (also called erasers: FTO, ALKBH5, and ALKBH3), and m^6^A-binding proteins (also called readers: IGF2BP1-3, YTHDF1-3, etc.) (5, 13). Compared to normal tissues, most m^6^A regulators demonstrated remarkable differential expression in TNBC patients (**Figure 1A**). We depicted the comprehensive landscape of m^6^A regulatory interactions, regulator connection, and their prognostic significance for TNBC patients using the m^6^A regulator network (**Figure 1B**). Importantly, based on survival analysis, among 26 m^6^A regulators, we found that YTHDF3 and IGF2BP1 overexpression showed poor prognosis, whereas RBM15B and HNRNPC overexpression demonstrated good prognosis (**Figure 1C**). By joint difference, survival, risk, and prognosis analysis, we screened out YTHDF3 as the only significant regulated gene (**Figure 1D**). Taken together, these data highlight the oncogenic role of YTHDF3 in TNBC progression.

### YTHDF3 promotes tumor progression in TNBC

**Figure 2:**
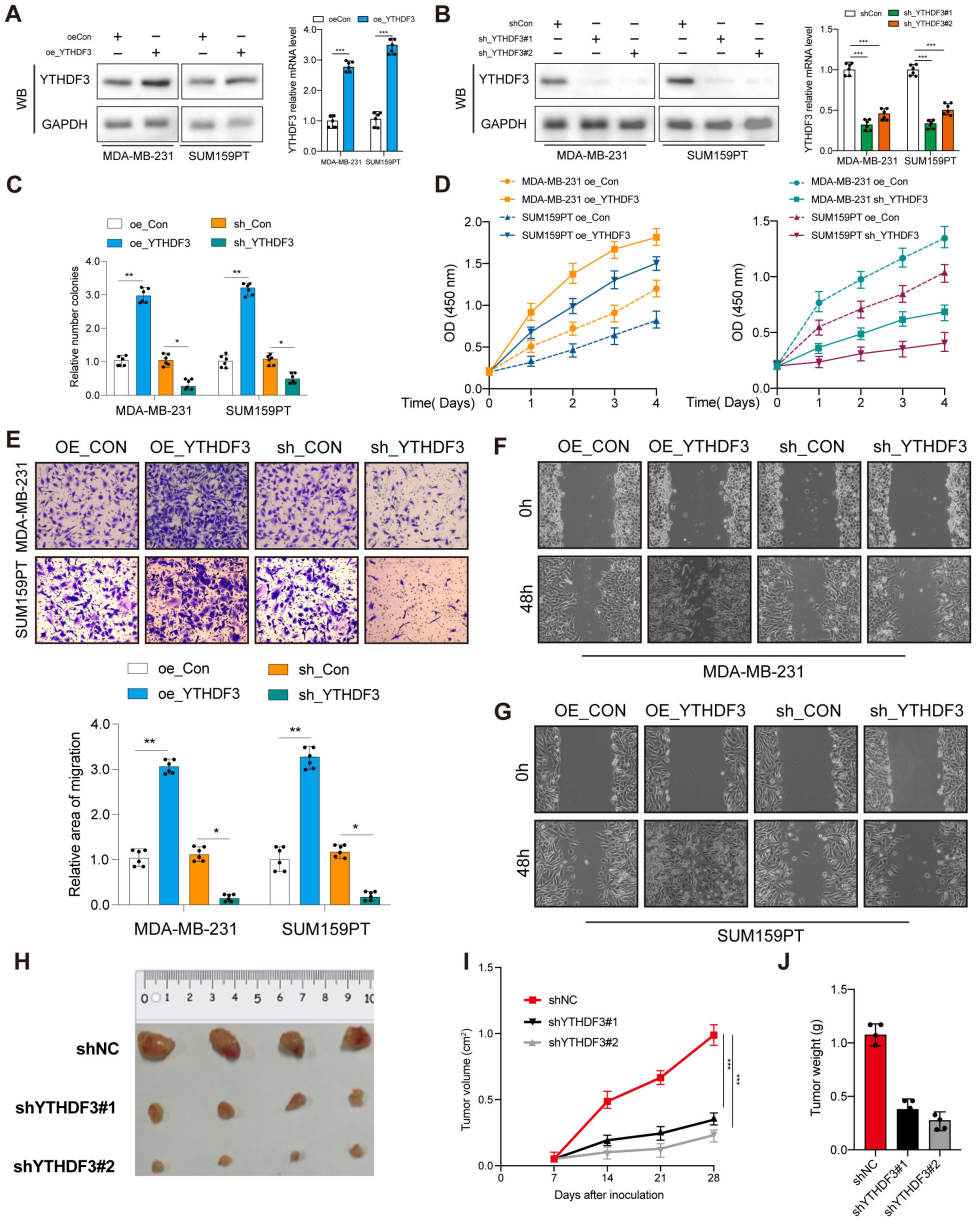
YTHDF3 promotes tumor progression in TNBC. **(A, B)** Transfection overexpression and knockdown efficiencies were validated by Western blotting and qPCR, respectively (n = 6). Data are mean ± SEM. ***p < 0.001. Two-tailed unpaired Student’s t-test. **(C)** Colony formation assay was used to analyze the proliferation viability of YTHDF3 in TNBC cells (n = 6). Data are mean ± SEM. *p < 0.05, **p < 0.01, compared with the control group. **(D)** CCK-8 was used to analyze the proliferation viability of YTHDF3 in TNBC cells (n = 6). Data are mean ± SEM. *p < 0.05, **p < 0.01, compared with the control group. **(E)** Transwell migration assay was used to analyze the migration viability of YTHDF3 in TNBC cells (n = 6). Data are mean ± SEM. *p < 0.05, **p < 0.01, compared with the control group. **(F, G)** Wound healing assay was used to analyze the migration viability of YTHDF3 in TNBC cells (n = 6). **(H)** The image of mice bearing subcutaneous tumors derived from SUM159PT cells treated with different treatments (shNC, shYTHDF3#1, or sh YTHDF3#2) at the indicated times. **(I)** The xenograft growth curves for the shYTHDF3#1, shYTHDF3#2, and shNC groups were plotted by measuring the tumor size (width2 × length × π/6) with a Vernier caliper every 7 days. **(J)** Nude mice were sacrificed, and xenografts were harvested and weighed. Data are mean ± SEM (n = 3). *p < 0.05, **p < 0.01, ***p < 0.001, compared with the control group. TNBC, triple-negative breast cancer.

Next, a range of functional experiments were used to explore the biological function of YTHDF3 in TNBC. YTHDF3 overexpression or knockdown was transfected in MDA-MB-231 and SUM159PT cells. Transfection efficiency was evaluated by Western blotting and qPCR (**Figures 2A, B**; **Supplementary Figure S1**). Colony formation assays and CCK-8 assays showed that the proliferative capacity and colony formation ability of the Vector-YTHDF3 group was significantly promoted, whereas YTHDF3 knockdown decreased cell proliferation and colony formation capacity compared to those in the control group (**Figures 2C, D**). In addition, our research verified that YTHDF3 overexpression markedly promoted the migration and invasion capabilities of TNBC cells (MDA-MB-231 and SUM159PT), while YTHDF3 knockdown decreased the migration and invasion capabilities compared to those in the control (**Figures 2E–G**). The SUM159PT cell suspension mixtures were used to establish an *in vivo* xenograft model. During the observation of flank xenografts in BALB/c nude mice for 28 days, YTHDF3 knockdown significantly delayed tumor progression, as the volume of YTHDF3-deficient tumors was significantly decreased compared with that of control tumors (**Figures 2H, I**). The nude mice were sacrificed, and the xenografts were harvested and weighed; the results showed that tumor weights were significantly reduced (**Figure 2J**). Therefore, these results suggested that YTHDF3 may influence the degree of malignancy in TNBC.

### Functional annotations of YTHDF3 in TNBC

**Figure 3:**
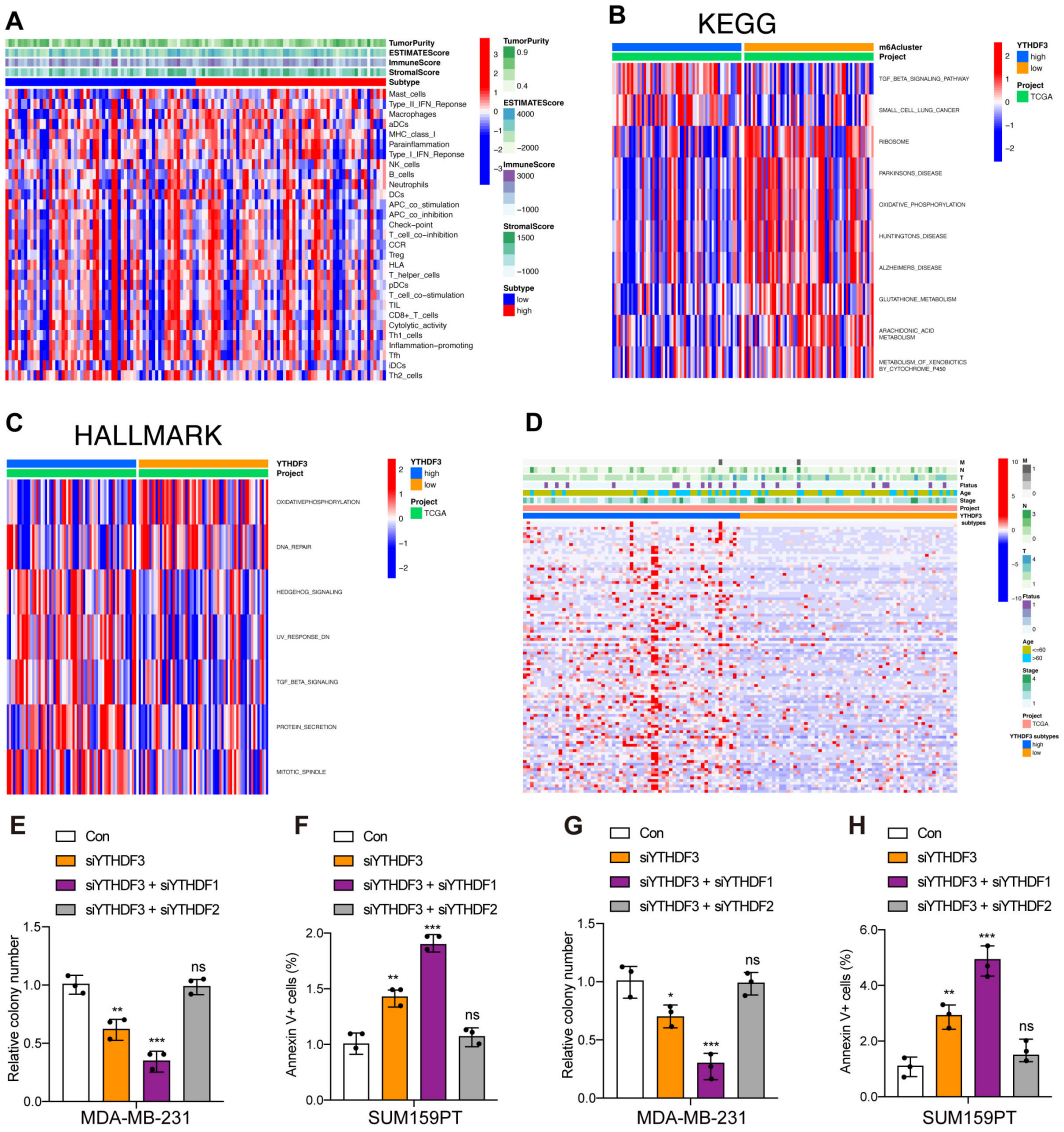
Functional annotations of YTHDF3 in TNBC. **(A)** Correlations of YTHDF3 expression with 29 immune-associated gene sets, immune score, stromal score, ESTIMATE score, and tumor purity. **(B)** Gene set variation analysis (GSVA) of YTHDF3 in TCGA cohort, as determined by Gene Expression Omnibus (red, high score; blue, low score); Kyoto Encyclopedia of Genes and Genomes (KEGG). **(C)** Gene set variation analysis (GSVA) of YTHDF3 in TCGA cohort, as determined by Gene Expression Omnibus (red, high score; blue, low score); HALLMARK. **(D)** Differences in clinical features (TNM, age, stage, and survival status) of TNBC between the low‐YTHDF3 and high‐YTHDF3 subtypes. **(E–H)** Cell viability **(E, G)** and cell apoptosis **(F, H)** in MDA-MB-231 and SUM159PT cells with different treatments. Data are mean ± SEM (n = 3). NS, non-significant; *p < 0.05, **p < 0.01, and ***p < 0.001, compared with the control group. TNBC, triple-negative breast cancer; TCGA, The Cancer Genome Atlas.

Immune cell infiltration analysis showed that high-YTHDF3 patients showed no significant enhancement compared to the low-YTHDF3 patients using the single-sample Gene Set Enrichment Analysis (GSEA) (ssGSEA) algorithm (**Figure 3A**), which suggested that YTHDF3 did not promote tumor progression by regulating the immune system. To explore the underlying molecular mechanisms of YTHDF3 in TNBC patients, we evaluated the biological function differing in the high-YTHDF3 and low-YTHDF3 subgroups of TNBC patients using GSVA, in which the results showed that the high-YTHDF3 patients were mainly related with the TGF-β signaling (**Figure 3B**), protein secretion, and mitotic spindle signaling pathways in TCGA dataset (**Figure 3C**). We evaluated the correlations between YTHDF3 expression and clinical properties in TCGA dataset; we screened the DEGs in the high- and low-YTHDF3 patients with TNBC (**Figure 3D**). Interestingly, we found that co-silencing YTHDF3/1 synergistically suppresses TNBC progression, while knockdown YTHDF3/2 rescues the inhibitory effect of knockdown YTHDF3 only (**Figures 3E–H**), in which interaction with other m^6^A regulators YTHDF1/2 were involved in the regulatory modification mechanism of YTHDF3.

### CENPI was identified as a downstream target of YTHDF3

**Figure 4:**
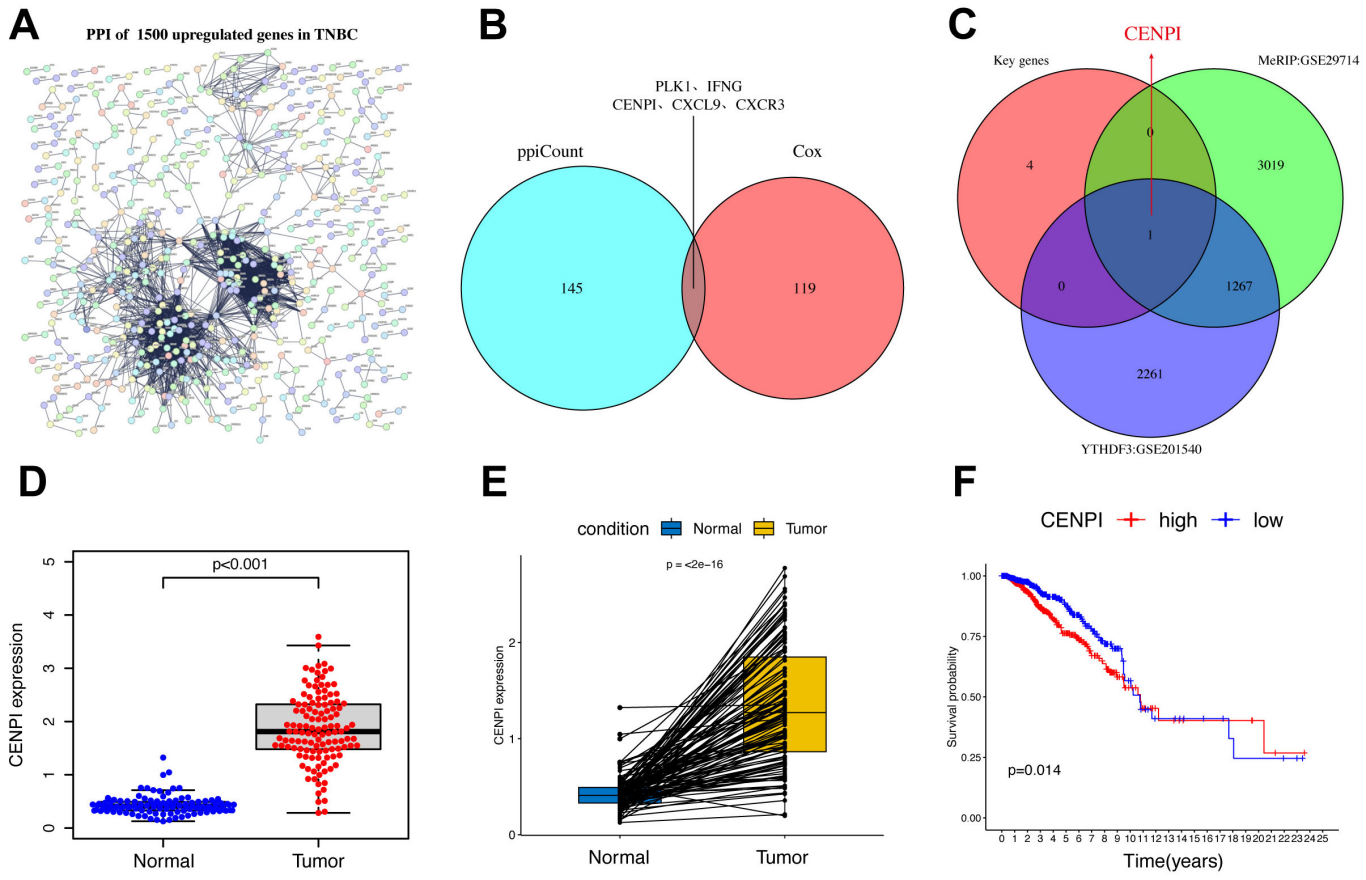
CENPI was identified as a downstream target of YTHDF3. **(A)** The PPI network with the STRING confidence score >0.8 based on 1,550 upregulated genes in TNBC. **(B, C)** The Venn plot shows five crucial genes (PLK1, IFNG, CENPI, CXCL9, and CXCR3) based on the 146 key genes of PPI network and 120 prognostic genes. **(C)** The Venn plot shows that CENPI was identified as a downstream target of YTHDF3 based on the crucial genes MeRIP (GEO: GSE29714), and YTHDF3-RIP (GEO: GSE201540). **(D)** The expression of DEPDC1 between normal and TNBC. **(E)** Pairwise difference analysis of DEPDC1 between normal and tumor. **(F)** Kaplan–Meier overall survival (OS) curves for tumor patients in accordance with CENPI expression in The Cancer Genome Atlas (TCGA) cohort. PPI, protein–protein interaction; TNBC, triple-negative breast cancer.

The limma package was used to screen 1,500 DEGs to investigate the potential biological behavior of YTHDF3 modification patterns between the high-YTHDF3 and low-YTHDF3 expression groups. STRING database (confidence value >0.8) was used to show the PPI network of the interactions among DEGs (**Figure 4A**). The five crucial genes (PLK1, IFNG, CENPI, CXCL9, and CXCR3) were based on the 146 key genes of the PPI network and 120 prognostic genes (**Figure 4B**). Then, CENPI was identified as a downstream target of YTHDF3 based on the crucial genes Methylated RNA immunoprecipitation sequencing (MeRIP) (GEO: GSE29714) and YTHDF3-RIP (GEO: GSE201540) (**Figure 4C**). Subsequently, the differential analysis showed that CENPI expression was significantly increased in TNBC compared with normal tissue using TCGA dataset (**Figures 4D, E**). The K-M analysis revealed that CENPI overexpression in tumor patients indicates poor survival (**Figure 4F**). Therefore, YTHDF3 may depend on m^6^A modification manner to regulate CENPI to play a carcinogenic role.

### YTHDF3 regulated CENPI expression in an m^6^A-dependent manner

**Figure 5:**
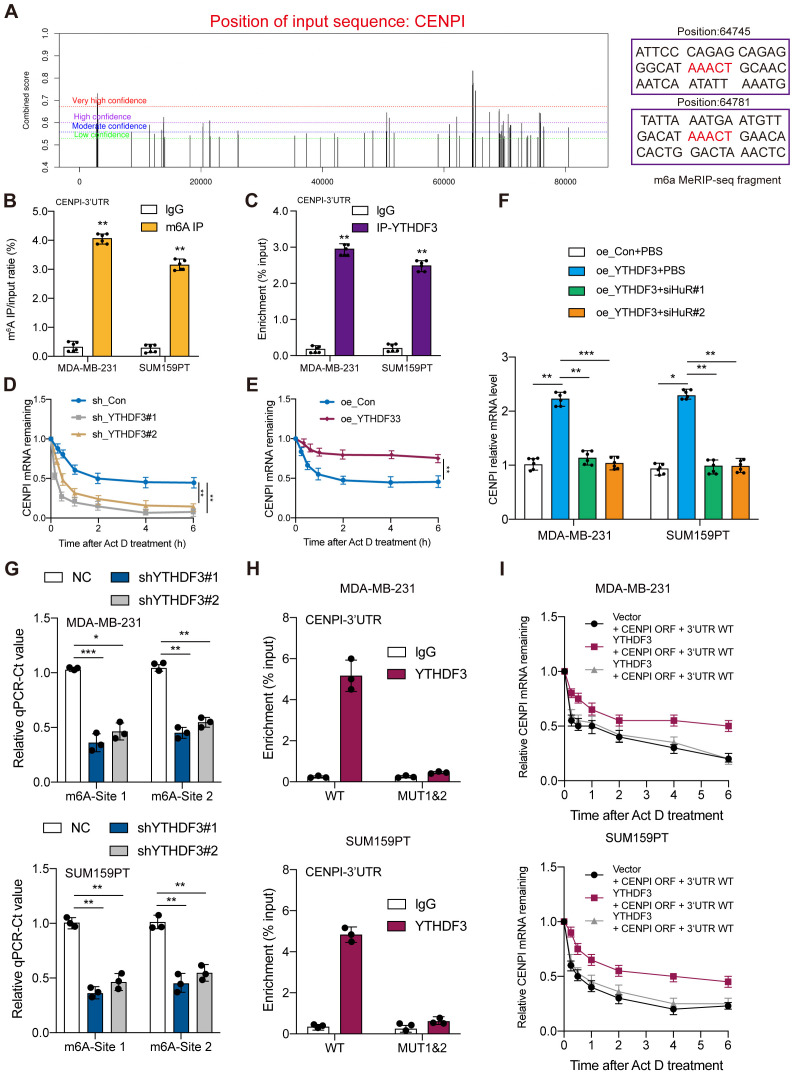
YTHDF3 regulated CENPI expression in an m^6^A-dependent manner. **(A)** The potential m^6^A sites in CENPI were predicted by SRAMP. The different colored lines indicate different confidence levels. **(B)** The mRNA of CENPI was enriched by the m^6^A-specific antibody compared to IgG in MDA-MB-231 and SUM159PT cells. **(C)** The mRNA of CENPI was enriched by the anti-YTHDF3 antibody compared to IgG in MDA-MB-231 and SUM159PT cells. **(D, E)** RT‐qPCR assay showing CENPI mRNA stability in MDA-MB-231 cells. **(F)** The effect of silencing HuR on CENPI expression in MDA-MB-231 and SUM159PT cells. **(G)** The threshold cycle (Ct) of qPCR shows SELECT results for detecting the m^6^A site in mRNA 3′UTR of CENPI in MDA-MB-231 and SUM159PT cells with or without knockdown of m^6^A regulators. **(H)** RIP‐qPCR detecting binding of YTHDF3 to the CENPI 3′UTR. **(I)** RT‐qPCR assay showing CENPI mRNA stability. Data are mean ± SEM (n = 3). *P < 0.05, **P < 0.01, ***P < 0.001, compared with the control group.

The m^6^A site prediction tool SRAMP was used to predict the distinct m^6^A sites in CENPI at single-based resolution (14) (**Figure 5A**). Then, MeRIP-RT–qPCR was performed to investigate whether gene expression affected m^6^A modification; the results indicated CENPI mRNA enrichment in the m^6^A-specific antibody (**Figure 5B**). RIP and RT–qPCR were used to evaluate RNA enrichment, and the results showed that the mRNA of CENPI was enriched by the anti-YTHDF3 antibody compared with IgG in the MDA-MB-231 and SUM159PT cell lines, which confirmed the direct interaction between YTHDF3 and CENPI (**Figure 5C**). Moreover, YTHDF3 overexpression was markedly prolonged (**Figure 5D**), while YTHDF3 knockdown obviously shortened the half-life of CENPI mRNA in TNBC cells (**Figure 5E**). Moreover, silencing mRNA stabilizer HuR dramatically diminished YTHDF3‐induced CENPI upregulation (**Figure 5F**), which demonstrated that YTHDF3 regulated CENPI expression by modulating its mRNA stability. Importantly, we found that the m^6^A modification of two potential nucleotide sites (Site1:64745 and Site2:64781) on the CENPI mRNA 3′UTR were validated by SELECT‐qPCR; the results showed that the m^6^A levels of the two sites on CENPI mRNA were dramatically decreased by YTHDF3 knockdown (**Figure 5G**). In addition, the association of YTHDF3 with the m^6^A‐modified region of CENPI mRNA could be abrogated by introducing mutations in its putative m^6^A‐modified site (AAACT-AGACT) (**Figure 5H**); the effects of IGF2BP3 on increasing BCAS2 mRNA stability were drastically abolished following the mutation of the specific m^6^A‐modified site (**Figure 5I**). Taken together, these data revealed that YTHDF3 mediated the degradation of CENPI mRNA by m^6^A modification.

### YTHDF3 promotes TNBC progression through CENPI expression

**Figure 6:**
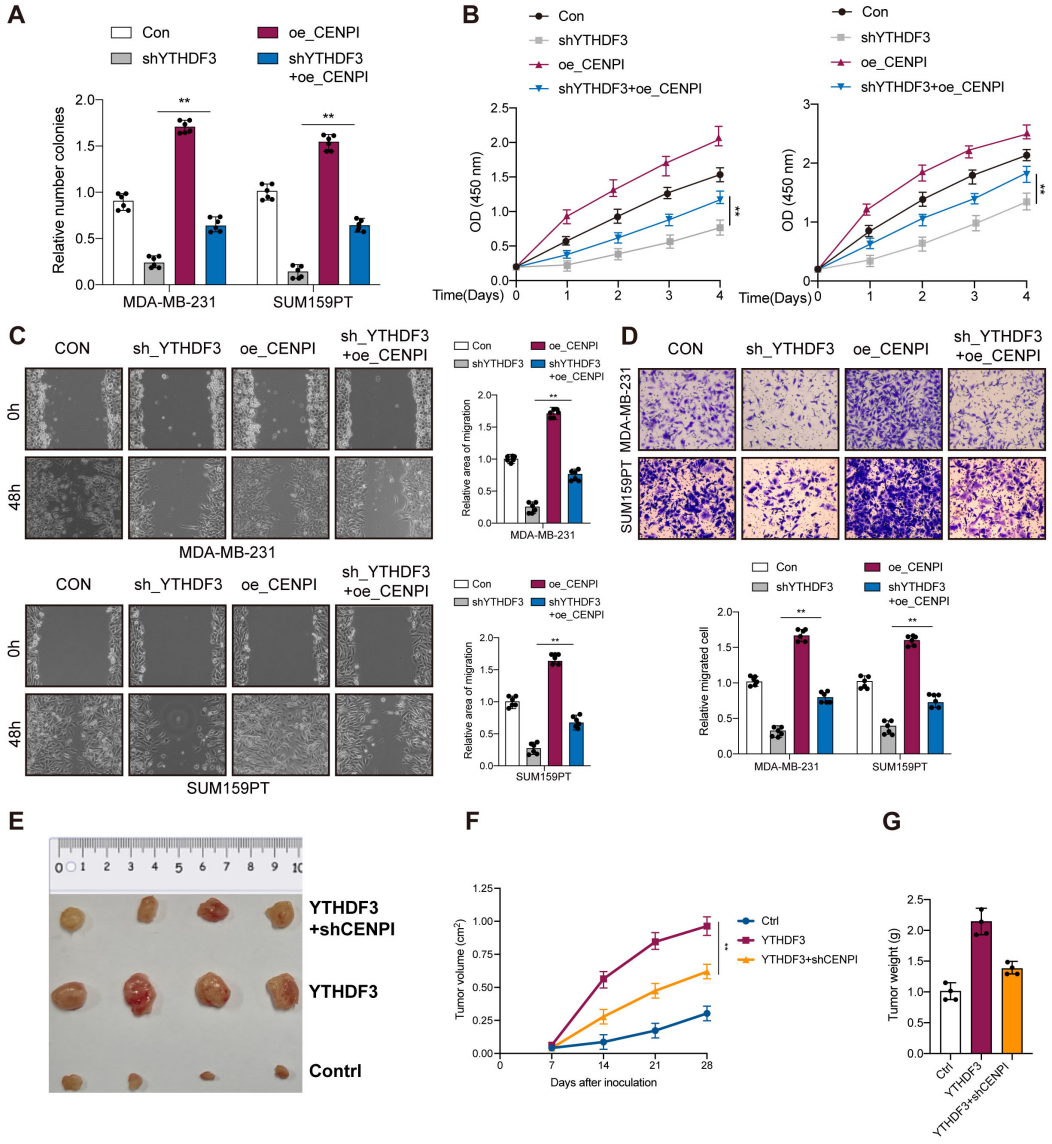
YTHDF3 promotes TNBC progression through CENPI expression. **(A)** Colony formation assay indicated the rescue effect of CENPI on YTHDF3 silencing in MDA-MB-231 and SUM159PT cells. **(B)** Cell viability was measured in YTHDF3 silencing cells with or without overexpression of CENPI in MDA-MB-231 and SUM159PT cells. **(C, D)** Transwell migration assays and wound healing assays were performed in YTHDF3-deficient cells with or without overexpression of CENPI in MDA-MB-231 and SUM159PT cells. All the data are presented as the mean ± standard deviation (n = 3). **p < 0.01, compared with the control group. **(E)** The image of mice bearing subcutaneous tumors derived from SUM159PT cells treated with different treatments (Ctrl, YTHDF3 overexpression, or YTHDF3 overexpression+ CENPI knockdown) at the indicated times. **(F)** The xenograft growth curves for the YTHDF3 overexpression, YTHDF3 overexpression + CENPI knockdown, and Ctrl groups were plotted by measuring the tumor size (width2 × length × π/6) with a Vernier caliper every 7 days. **(G)** Nude mice were sacrificed, and xenografts were harvested and weighed. Data are mean ± SEM (n = 3). **p < 0.01, compared with the control group. TNBC, triple-negative breast cancer.

As expected, overexpression of CENPI also promoted cell proliferation, colony formation, and migration in MDA-MB-231 and SUM159PT cells, confirming the oncogenic effects of CENPI (**Figures 6A–D**). Since YTHDF3 could regulate CENPI expression, rescue experiments were carried out to verify the interaction between YTHDF3 and CENPI in TNBC progression. CENPI overexpression could partially counteract the antitumor effects on cell viability (**Figure 6A**), colony formation (**Figure 6B**), and migration (**Figures 6C, D**) mediated by shYTHDF3. Importantly, in the BALB/c mouse model, YTHDF3 overexpression-induced tumor progression could be partially abrogated by CENPI knockdown (**Figures 6E–G**). Collectively, YTHDF3 promotes TNBC progression through CENPI expression.

## Discussion

m^6^A modification is dynamically and reversibly regulated by m^6^A “writers”, “erasers”, and “readers”. An increasing number of studies have demonstrated that m^6^A editing promotes tumor development and progression (15, 16). Accumulating evidence has indicated that YTHDF3 promotes tumor progression including breast cancers (17), small-cell lung cancer (SCLC) (18), CRC (19), and other types (20–22); however, the role of YTHDF3 in TNBC is not well understood.

In this study, among these m^6^A regulators, we first identified the key m^6^A regulator YTHDF3 in TNBC using TCGA dataset through differential analysis, correlation analysis, Cox regression analysis, and survival analysis. We confirmed bioinformatically that the YTHDF3 expression was significantly higher in TNBC tumor samples than in normal samples. Moreover, the TNBC patients with high YTHDF3 expression had a worse prognosis. In *in vitro* experiments, we demonstrated that YTHDF3 promoted tumor progression in TNBC by CCK-8, colony formation assay, Transwell migration assay, and wound healing assay.

Next, we further explored the function of YTHDF3 in TNBC. Surprisingly, high-YTHDF3 patients had no obvious immune activity compared to low-YTHDF3 patients. In addition, GSVA showed that high-YTHDF3 patients were mainly related to the TGF-β signaling, protein secretion, and mitotic spindle signaling pathways.

YTHDF3, as regulator (“reader”) of m^6^A modification, has been reported to play an important role in tumor progression. For example, YTHDF3 facilitated HDAC6 translation, ultimately attenuating cell growth and cervical cancer development (23). YTHDF3 suppressed PFKL mRNA degradation via m^6^A modification, subsequently promoting tumor growth and lung metastasis of HCC cells (24). However, the mechanism of the m^6^A modification of YTHDF3 in TNBC is still unclear. To explore whether YTHDF3 regulated the progression of TNBC cells in an m^6^A-dependent manner, the Gene Expression Omnibus (GEO) database was used to explore the modification target gene of YTHDF3. Then, CENPI was identified as a downstream target of YTHDF3. CENPI belongs to the constitutive centromere-associated network, which regulates chromosomal segregation and alignment as well as guarantees a suitable mitosis process (10, 25–27). CENPI has been shown to affect tumorigenesis. The pan-cancer analysis demonstrated that CENPI is a potential diagnostic and prognostic biomarker in various cancers including TNBC (9). In addition, CENPI was related to immune cell infiltration and drug sensitivity in pan-cancer and can act as a potential treatment target to cure cancer patients (8).

We confirmed bioinformatically that CENPI was significantly upregulated in TNBC compared with normal tissue and that CENPI overexpression showed poor survival. MeRIP-RT–qPCR demonstrated that YTHDF3 modulated CENPI mRNA stability via m^6^A modification. Importantly, *in vitro* experiments and rescue experiments were carried out to verify whether YTHDF3 promotes TNBC progression through CENPI expression. The above data suggested that the YTHDF3–CENPI axis has an important role in TNBC progression and provides a novel potential prognostic biomarker for TNBC.

## Conclusion

In conclusion, our study has illustrated that YTHDF3-mediated m^6^A modification in CENPI mRNA stabilization contributes to the tumorigenesis and poor prognosis of TNBC, providing a distinct mechanistic insight in m^6^A‐dependent, which should be helpful for developing CENPI signaling‐targeted inhibitors.

## Data Availability

The original contributions presented in the study are included in the article/**Supplementary Material**. Further inquiries can be directed to the corresponding author.

## Supplementary material

Supplementary Table 1 26 m^6^A regulators.

Supplementary Table 2 The number of nodes of Protein–protein interaction network.

Supplementary Table 3 Cox analysis of the 1500 DEGs.
