# Supplementary material for: The m6A reader YTHDF3 promotes TNBC progression by regulating CENPI stabilization
Source: Front Oncol. 2025 May 8;15:1546723. doi: 10.3389/fonc.2025.1546723 (PMC12095154; doi:10.3389/fonc.2025.1546723)
Supplement: Supplementary file 2 [file DataSheet1.docx]

**
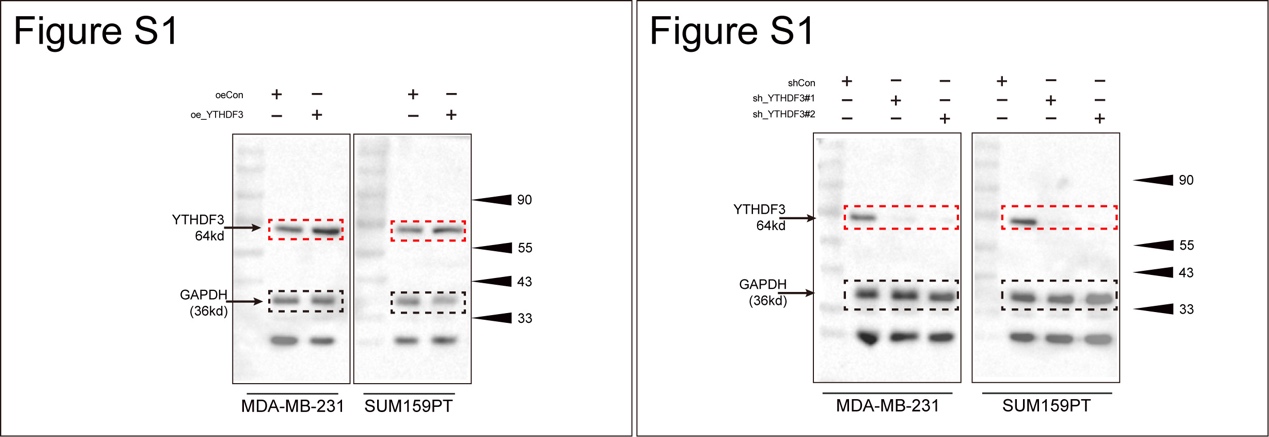
Supplementary Figure S1. Uncropped blots.**

Uncropped images of all blots shown in main and supplementary figures. Molecular weight markers are indicated on the left side of each blot, whereas predicted molecular weight of the protein of interest indicated by an arrow on the right.
